# The impact of COVID-19 on households´ income in the EU

**DOI:** 10.1007/s10888-021-09485-8

**Published:** 2021-06-01

**Authors:** Vanda Almeida, Salvador Barrios, Michael Christl, Silvia De Poli, Alberto Tumino, Wouter van der Wielen

**Affiliations:** 1 European Commission, Joint Research Centre (JRC-Seville), Edificio Expo, Calle Inca Garcilaso, 3, 41092 Seville, Spain; 2 Institute for Social and Economic Research (ISER), University of Essex, Wivenhoe Park, Colchester, Essex, UK

**Keywords:** COVID-19, Fiscal policy, Earnings subsidies, Income distribution, Unemployment

## Abstract

This analysis makes use of economic forecasts for 2020 issued by the European Commission in Autumn 2019 and Spring 2020, and of a counterfactual under a no-policy change assumption, to analyse the impact of the COVID-19 crisis on EU households´ income. Additionally, our analysis assesses the cushioning effect of discretionary fiscal policy measures taken by the EU Member States. We find that the COVID-19 pandemic is likely to affect significantly households’ disposable income in the EU, with lower income households being more severely hit. However, our results show that due to policy intervention, the impact of the crisis is expected to be similar to the one experienced during the 2008–2009 financial crisis. In detail, our results indicate that discretionary fiscal policy measures will play a significant cushioning role, reducing the size of the income loss (from −9.3% to −4.3% for the average equivalised disposable income), its regressivity and mitigating the poverty impact of the pandemic. We conclude that policy interventions are therefore instrumental in cushioning against the impact of the crisis on inequality and poverty.

**Supplementary Information:**

The online version contains supplementary material available at 10.1007/s10888-021-09485-8.

## 1 Introduction

Preliminary indicators on job destruction and unemployment benefits claims across European Union (EU) countries suggest that the impact of the COVID-19 pandemic on households will

✉ Michael Christl
Michael.CHRISTL@ec.europa.eu

[1] European Commission, Joint Research Centre (JRC-Seville), Edificio Expo, Calle Inca Garcilaso, 3, 41092 Seville, Spain

[2] Institute for Social and Economic Research (ISER), University of Essex, Wivenhoe Park, Colchester, Essex, UK

likely be exceptionally high (OECD 2020). Early findings suggest that the risk of job loss is highest in southern Europe and France (Doerr and Gambacorta 2020). At the country level, young, low-educated, low-income workers appear to face the highest income and employment risk (see Pouliakas and Branka (2020) for the EU; Galasso (2020) for Italy; Adams-Prassl et al. (2020) for Germany; Bradley et al. (2020) for the UK; Beland et al. (2020); Cajner et al. (2020); Cho and Winters (2020); Mongey et al. (2020) and Shibata (2020) for the US; and Aum et al. (2020) for South Korea).[1] For example, Joyce and Xu (2020) find that low earners in the UK are seven times as likely as high earners to have worked in a sector shut down following the lockdown. The consequences of the COVID-19 crisis on households´ income in particular, although unknown with precision yet, raise serious concerns.

Policies aimed at protecting those most directly hit by the crisis, either through discretionary measures (e.g., income subsidies or tax rebates), or through automatic stabilisation (e.g., unemployment benefits or lower taxes paid as a result of job loss and/or decrease in market incomes), could partly reduce the toll on household income and consumption.[2] Recent evidence from Denmark (Bennedsen et al. 2020), for instance, shows that without government support, in the form of labour subsidies, firms are expected to have proceeded with layoffs instead. In addition, Chetty et al. (2020) find that stimulus payments to low-income households in the US sharply increased consumer spending.[3]

The existing evidence suggests that the COVID-19 crisis will lead to an increase in both poverty and wage inequality in all European countries. Palomino et al. (2020), for instance, estimate the Gini coefficient to increase 2.2% in Europe. Moreover, historic data suggest that past events of this kind, even though much smaller in scale, have led to significant, persistent increases in the net Gini coefficient (by 1.25% five years after the pandemic) and raised the income shares of higher income deciles (Furceri et al. 2020). Therefore, a micro-based distributional analysis of the COVID-19 crisis and the cushioning fiscal policies taken in the crisis' aftermath – like the one done by Figari and Fiorio (2020) for Italy, Bronka et al. (2020) and Brewer and Tasseva (2020) for the UK and O'Donoghue et al. (2020) and Beirne et al. (2020) for Ireland – covering the whole EU is warranted to inform policy decisions in the EU.

This paper provides an assessment of the potential impact of discretionary fiscal policy measures adopted in the wake of the COVID-19 crisis on household income, poverty and inequality in the EU in 2020. The analysis is based on three macroeconomic scenarios. We use the European Commission (EC) Autumn 2019 Economic Forecast for 2020 to proxy the 2020 EU economy in absence of the COVID-19 pandemic ("no-COVID-19" scenario). The EC Spring 2020 Economic Forecast for 2020 represents a COVID-19 scenario that embeds the impact of discretionary policy measures taken or announced by governments, including those financed thanks to EU support, following the COVID-19 outbreak. This scenario also embeds

[1] Two groups moreover seem relatively more affected by the COVID-19 crisis vis-à-vis the Global financial crisis (Shibata 2020): (i) Women were about one third more likely to work in a sector that is now shut down (Joyce and Xu 2020); and (ii) Non-whites (see, e.g., Beland et al. (2020), Cho and Winters (2020), Cowan (2020) and Fairlie et al. (2020)), and immigrants (see, e.g., Borjas and Cassidy (2020) in the US. Platt and Warwick (2020) document a similar vulnerability of Pakistanis and Bangladeshis in the UK.)

[2] Nevertheless, many individuals would remain vulnerable if ensured 50% of their gross privately earned income (Midões 2020). In the EU, at least 99 million individuals live in households that cannot cover for two months of the most basic expenses only from their savings in bank accounts.

[3] The authors find none to modest short-run employment effects. One possible explanation is that firms have almost entirely stopped posting new vacancies; see, e.g., Costa Dias et al. (2020) for the UK and Campello et al. (2020) for the US. Similarly, many of those losing their jobs are currently also not (yet) looking for new ones (Coibion et al. 2020).

the role of automatic stabilisers, reflecting the existing features of each Member State's tax and transfer system ("COVID-19 including policy changes" scenario). We then construct a counterfactual 2020 macroeconomic scenario describing the effect of the COVID-19 pandemic without discretionary fiscal policy measures ("COVID-19 no policy changes" scenario). This scenario is based on estimates of the budgetary impact of the discretionary measures (taken from the Stability and Convergence Programmes submitted by the EU Member States) together with estimates on spending and revenue fiscal multipliers taken from the literature.

We use EUROMOD, the EU microsimulation model, to simulate and compare households' income, inequality and poverty indexes under each macroeconomic scenario and to estimate the overall impact of the crisis and the cushioning effect of discretionary fiscal measures. EUROMOD is a static tax-benefit microsimulation model covering in a comparable way all the EU Member States. The model enables consistent EU-wide tax-benefit and distributional analyses. We overcome the methodological challenge posed by the lack of up-to-date survey data by reweighting the microdata underlying EUROMOD based on the European Union Statistics on Income and Living Conditions (EU-SILC). The approach, somewhat similar to O'Donoghue et al. 2020; and Beirne et al. 2020, allows us to modify the population structure of the survey microdata and precisely mimic the aggregate employment, unemployment and total wage compensation – including wage compensation schemes – figures from each macroeconomic scenario.

Our main findings are threefold. First, our simulations show that the COVID-19 crisis is likely to have a significant impact on EU households' disposable income, leading to an average reduction of 9.3% in equivalised disposable income in absence of discretionary policy changes by EU Member States. Second, our results show that the impact of the COVID-19 crisis is expected to be highly regressive, with the lowest deciles of the income distribution being more severely hit, and lead to a significant increase in poverty. Third, the discretionary fiscal policy measures taken by the EU Member States in the crisis' aftermath have been instrumental in cushioning against the early impact of the crisis on households' income, inequality and poverty. When accounting for these measures, the average loss in equivalised disposable income is 4.3% and the increases in inequality and poverty are largely offset. To put our results into perspective, we also provide a direct comparison of the simulated impact of the COVID-19 crisis to the impact of the 2008–2009 financial crisis. Our results suggest that the social impact of the current crisis in the absence of policy intervention will be much larger than the one of the 2008/2009 crisis, however, due to policy intervention we expect a similar impact than observed in 2008/2009.

The rest of the paper is organised as follows. Section 2 describes the methodology used in the analysis. Section 3 presents the main findings. Section 4 concludes and discusses some policy implications.

## 2 Methodology

### 2.1 Macroeconomic Scenarios

We consider three alternative macroeconomic scenarios for the year 2020: First, we use the macroeconomic scenario for 2020 included in the EC Autumn 2019 Economic Forecast and in the EC Spring 2020 Economic Forecast to proxy the macroeconomic conditions of, respectively, a 2020 in which COVID-19 did not happen ("No-COVID-19" scenario) and a 2020

incorporating the effect of the COVID-19 pandemic and of the related policy response, including discretionary policy measures ("COVID-19 including policy changes" scenario).

Second, we consider a counterfactual "COVID-19 no policy-changes" scenario for 2020 to gauge the effect of policy measures taken by EU countries to cushion the impacts of the COVID-19 crisis. This counterfactual no policy-change scenario is derived by estimating GDP growth and changes in employment if no discretionary policy measures had been taken to mitigate the socio-economic consequences of the COVID-19 pandemic, and only automatic stabilisers would be at play. The construction of the no policy-change scenario requires some steps and information (explained in detail below): the EC Spring 2020 Economic Forecast of GDP growth, the expected budgetary impact of COVID-19 related discretionary fiscal policy measures, the associated fiscal multipliers (and their evolution throughout 2020) and the GDP-employment relationship by sector and Member State.

In our setting, a comparison of the "No-COVID-19" and the "COVID-19 including policy changes" scenarios allows deriving information on the estimated impacts of the COVID-19 crisis, including the shutdown of major parts of the economy, as well as policy measures taken by Member States to counteract the strong impact of the pandemic. As a note of caution, it should be noted that differences between forecasts could potentially be affected by factors other than COVID-19, for example the availability of more up-to-date information of the economy. We are, however, confident that most of the differences observed can be attributed to the impact of COVID. In addition, it should be mentioned that the assumptions underlying the EC Spring Forecast 2020 are crucial for the results of our analysis. In particular, the EC Spring 2020 Economic Forecast estimated a collapse of economic output in the first half of 2020 while it was assumed that, after that, the containment measures would be gradually lifted, and the pandemic remained under control. An additional assumption was that both, monetary and fiscal measures within the EU would effectively cushion the immediate economic impact of the COVID crisis and therefore limit the immediate damage on the economy.

Similarly, a comparison of the "No-COVID-19" and the counterfactual "COVID-19 no policy changes" scenarios allows us to derive the estimated impact of COVID-19 in absence of discretionary policy measures, consequently enabling us to evaluate the cushioning effect of these measures.

For all these scenarios, the impact of changes in macroeconomic variables on households' incomes are analysed by reweighting the baseline 2019 EUROMOD simulations, applying the predicted changes in several target variables, among them employment, unemployment and total wage compensation. The reweighting procedure is described in detail in the following section.

To construct the counterfactual no policy-change scenario, we start by estimating the GDP growth that would be observed in the absence of policy measures. We do this by removing the expected economic effect of COVID-19 related discretionary fiscal policy measures from the EC Spring 2020 Economic Forecast for GDP growth in 2020, in three main steps. The first step involves obtaining estimates of the budgetary impact of COVID-19 related discretionary fiscal policy measures. For national spending and revenue measures, we use the 2020 Stability and Convergence Programmes (SCP) submitted by the EU Member States.[4] In the same vein, we include EU-funded public spending through the European Structural and Investment Funds (ESIF) and the Coronavirus Response Investment Initiative (CRII), since they make up a

[4] The only exception is the Netherlands, since the Dutch Stability Programme did not report any estimates of COVID-19 related measures. Instead, data were taken from the Netherlands Bureau for Economic Policy Analysis (CPB) June projections.

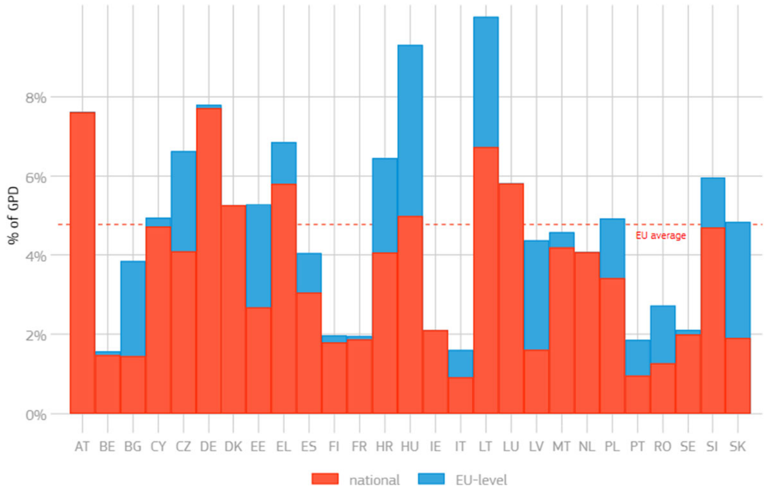

**Fig. 1** COVID-19 related discretionary fiscal measures with a budgetary impact in 2020. Note: includes spending and revenue measures and EU-level spending, excludes guarantees significant share of spending measures in some Member States.[5] The exact amounts for the EU-funded spending by Member States were obtained from the EC's Directorate-General for Budget. Loans and guarantees ensuring businesses' liquidity are disregarded as either they have no direct budgetary impact, or their economic impact is highly uncertain. A summary of the budgetary impact of all the discretionary fiscal policy measures removed in the counter-factual scenario is presented in Fig. 1.[6]

It is important to note that the counterfactual scenario solely corrects for discretionary fiscal measures taken in response to the pandemic, which include only newly introduced policies or significant changes to existing policies. For example, for countries that already had wage compensation schemes in place before the crisis (e.g., the Cassa Integrazione in Italy) and did not change the scheme significantly, the automatic changes in spending with these schemes is not included in Fig. 1. Similarly, some countries reported large volume effects in the existing unemployment scheme (e.g., Belgium), which are not included. Therefore, the no policy-change counterfactual does not allow for conclusions on the impact of automatic stabilisers. Nevertheless, we follow this approach for two important reasons. First, using one, heavily standardised, source (cf. the SCPs) allows for the best possible consistent comparison across countries of our final results. Second, since the counterfactual builds on the EC Spring 2020 Economic Forecast, it is suitable to rely on the same set of information used to construct said forecast.

[5] As this mainly concerns a re-orientation of existing EU funds – in contrast to the future recovery package – their distribution across Member States follows the existing agreements, with a focus on newer Member States.

[6] A detailed overview of all the national, COVID-related discretionary spending and revenue measures included in the analysis to the online appendix. The online appendix also includes an overview by Member State of the EU-funded public spending through the ESIF and the CRII.

[7] For example, Gechert (2015) provides a comprehensive meta-analysis of spending and revenue multipliers estimated using both macroeconometric models as well as structural models. Coenen et al. (2012) reconcile the fiscal multipliers from seven prominent structural policy models, including those by the European Central Bank (NAWM), the EC (QUEST), the IMF (GIMF) and the OECD (OECD Fiscal). Kilponen et al. (2019), in their turn, look across the dynamic macro models used by the member institutions of the European System of Central Banks. Finally, van der Wielen (2020) documents recent, empirical estimates, with a particular focus on the EU.

The second step involves obtaining estimates of fiscal multipliers, using results from well-established academic contributions, in order to obtain a set of representative small, average and large multipliers.[7] For the revenue measures we employ the mean multiplier found in the meta-analysis (0.54) by Gechert (2015), as it appears to be a good trade-off between the multipliers at the lower end of the spectrum (e.g., 0.04 for labour income taxes in the EMU using DSGE modelling by Kilponen et al. 2019) and the multipliers at the higher end (e.g., 1.39 for the average revenue multiplier of the narrative studies listed in Table 1 of van der Wielen 2020). On the spending side we rely on a multiplier of 0.9. This is closely in line with the multiplier in the European Commission's QUEST model underlying the forecasts, where the multiplier is smaller, but close to 1. The meta-analysis by Gechert (2015) moreover also finds a mean government spending multiplier around 1. Kilponen et al. (2019), in turn, find an average multiplier of 0.93 for temporary government consumption. Finally, the data on the discretionary measures gathered show that on average 77% of all measures are spending measures and only 23% are revenue measures. Therefore, the resulting average weighted multiplier for the overall budget balance (0.82) is closely in line with, although slightly above, the average multiplier used in the recommendations under the EU's excessive deficit procedure in the recent past.[8] Using the annual values for the multipliers obtained from the literature, we estimate quarterly values, by making assumptions on the intensity of the impact of policy measures in each stage of the observed/expected progression of lockdown measures. We consider three scenarios for the quarterly evolution of the multipliers, a low, a medium and a high scenario. The multipliers considered in each quarter in each of the three scenarios are presented in Fig. 2.

We start from an average multiplier in the first quarter of 2020 as no or little lockdown measures were in place. In the second quarter, the multiplier is assumed to drop considerably in the low scenario, slightly drop in the medium scenario and stay the same in the high scenario, reflecting different possibilities for the severity of the impact of lockdown measures. The impact of discretionary fiscal measures is then expected to strengthen in the third quarter, as lockdown measures are lifted and a possible overshooting of consumption may be observed, with a small increase in the multiplier in the low scenario, a big increase in the medium scenario, and a very big increase in the high scenario. Finally, the situation is assumed to reverse to values closer to the average in the fourth quarter, staying exactly on average in the low scenario, slightly above average in the medium scenario and well above average in the high scenario.

The third step involves multiplying the estimated budgetary impact of the discretionary budget measures (as a percentage of GDP) by the estimated fiscal multipliers, to obtain the expected GDP impact of these measures, for each of the three multiplier scenarios, low, medium and high.[9] The result of this multiplication is then subtracted from the EC's Spring

[7] For example, Gechert (2015) provides a comprehensive meta-analysis of spending and revenue multipliers estimated using both macroeconometric models as well as structural models. Coenen et al. (2012) reconcile the fiscal multipliers from seven prominent structural policy models, including those by the European Central Bank (NAWM), the EC (QUEST), the IMF (GIMF) and the OECD (OECD Fiscal). Kilponen et al. (2019), in their turn, look across the dynamic macro models used by the member institutions of the European System of Central Banks. Finally, van der Wielen (2020) documents recent, empirical estimates, with a particular focus on the EU.

[8] As documented by Górnicka et al. (2020), the average multiplier between 2012 and 2015 amounted to 2/3.

[9] Throughout the analysis it is assumed that the discretionary policy measures included take place for 40%, 50% and 10% in quarters 2, 3 and 4, respectively. This is not unimaginable since it concerns measures taken in the weeks and months after the crisis first hit the economy. The extension of these measures to later quarters or the introduction of additional measures was not yet covered. Combining this information with the multipliers recorded in Figure 2, this leads to a yearly multiplier close to 1 in the medium no policy-change scenario. The corresponding yearly multipliers in the low and high scenario are 0.56 and 1.53, respectively.

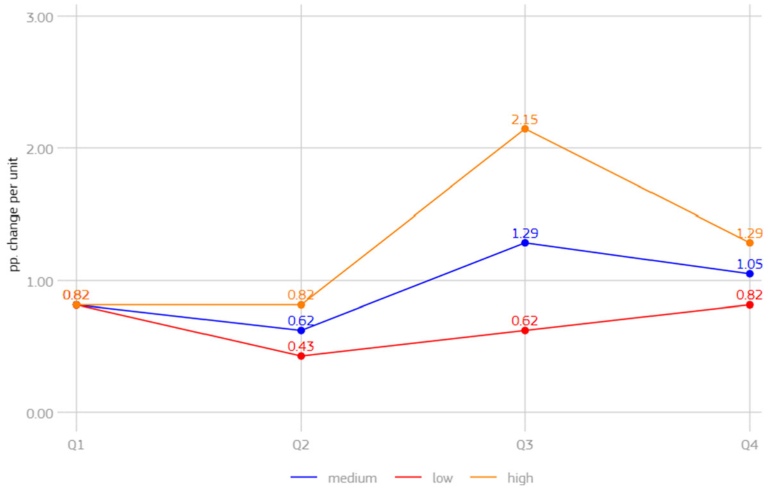

**Fig. 2** Budget balance multipliers used for 2020 no policy-change scenarios

2020 Economic Forecast for GDP growth, resulting in three no policy-change scenarios for GDP growth, as summarised in Fig. 3. As can be seen, discretionary policy measures are expected to play a considerable role in mitigating the recessionary impact of the pandemic, with GDP growth for the EU average being between 1.4 and 3.5 percentage points lower in the no policy-change scenario.

Finally, the estimated GDP growth values are translated into an expected impact on employment (expressed in comparison to employment changes reported in the EC Autumn 2019 Economic Forecast). The Trade-SCAN model – see, e.g., Román et al. (2019) – is used twice to do this. The Trade-SCAN model is a multi-country input-output model using OECD

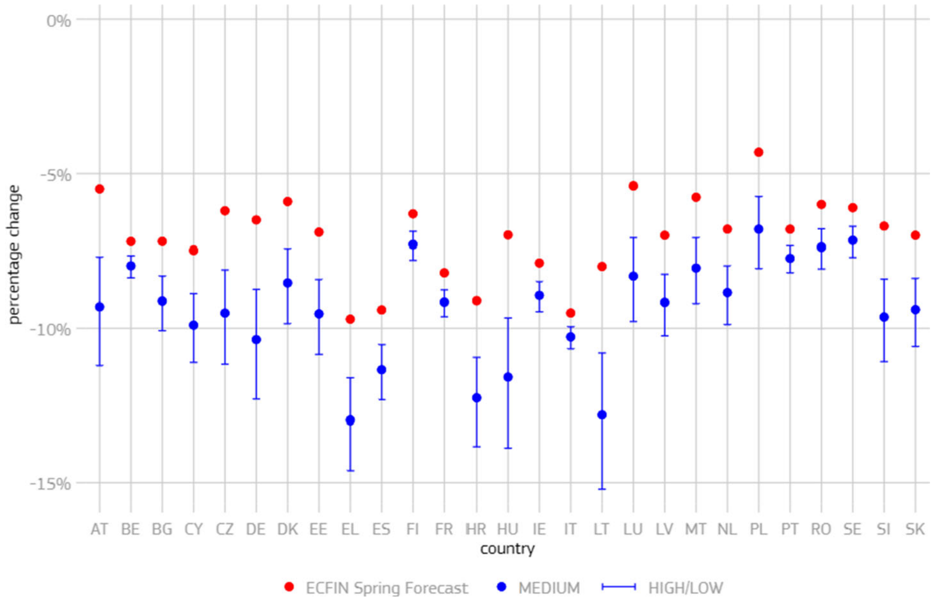

**Fig. 3** 2020 GDP growth - EC Spring 2020 Economic Forecast vs. counterfactual no policy-change scenarios employment and Inter-country Input-Output Tables (edition 2018) for 2015. It allows us to translate GDP shocks via the value-chain on sector level. In a second step, Trade-SCAN can estimate employment at risk that is associated directly and indirectly to consumption and investment. First, the whole economy GDP estimates are brought to the sectoral level using the latest sectorial distribution estimated in Trade-SCAN, e.g., accounting for the relatively large burden of the overall shock on sectors such as “Accommodation and food services”. Second, using the GDP-employment relationship by sector inherent in the Trade-SCAN model, the sectoral GDP changes are translated into sectoral employment changes. Lastly, the sectoral employment changes are aggregated to obtain aggregate employment changes for each Member State. The results for the aggregate employment changes in each of the scenarios, together with the employment changes considered in the EC Spring 2020 Economic Forecast can be seen in Table A2 in the Annex, together with a depiction of the relationship between GDP growth and employment changes underlying the estimates in the different scenarios in Fig. A1. As a note of caution, it has to be emphasised that in the counterfactual no-policy change scenario employment changes are derived from GDP growth forecast, while in the other two scenarios the changes in employment are taken directly from the forecast, i.e., not requiring any intermediate steps. Therefore, it cannot be ruled out that the aforementioned methodology for constructing the no policy-change scenario introduces additional discrepancy between the scenarios. For example, expert judgement may have been applied to the forecasts; something that could not be corrected due to a lack of information on such adjustments. Nevertheless, as illustrated in Fig. A1, the employment changes constructed using the abovementioned methodology are largely in line with the historically observed relationship between GDP growth and percentage changes in employment in the EU.

## 2.2 EUROMOD

Our analysis makes use of EUROMOD, the EU microsimulation model, version I2.0+. Making use of representative survey data from the European Union Statistics on Income and Living Conditions (EU-SILC), the model is a static tax-benefit calculator, designed to provide results which are representative at the country-level and validated against aggregate national statistics. The model simulates the personal income taxes, social benefits and social security contributions in all EU countries and can be used to study the effect of actual and perspective policy reforms on household incomes, inequality, poverty and the government’s fiscal balance.[10]

The scope of EUROMOD simulations includes direct taxes and non-contributory benefits in place in each country. Some contributory benefits, such as unemployment benefits, are also simulated in most countries, making assumptions on working history for eligibility purposes where needed. In countries where simulations of unemployment benefits are not satisfactory, the value recorded in the underlying EU-SILC data is used instead.

We apply reweighting to replicate in EUROMOD the macroeconomic conditions of the scenarios of interest. We make use of EUROMOD simulations based on data from 2017 EU-SILC and 2019 policy systems (see Annex 4 for further details on this approach).[11] Non-simulated tax-benefit instruments are uprated to their 2019 values making use of specific

[10] For a detailed description of EUROMOD and of the scope of its simulation see Figari and Sutherland (2013).
[11] In a number of countries, the national version of SILC has been used either directly or to complement the information contained in the EU-SILC UDB distributed by EUROSTAT.

uprating factors (see EUROMOD Country Reports 2019 for more information on the data used and uprating factors).[12]

Unfortunately, detailed policy modelling for 2020 and COVID-19 related policies was not available when this analysis was carried out. Consistent with the EC Spring 2020 Economic Forecast, this paper takes into account the impact of Short-Time Work (STW) Schemes on wages through reweighting. For future research, however, better data and detailed policy modelling might help to get a more detailed picture on the impact of COVID-19 on the EU Member States.

It is important to note that EUROMOD is a static model, i.e., it only measures the impact of policy and income changes without making assumptions about behavioural effects. Given that our analysis focuses on the immediate consequences of the COVID-19 crisis, this seems a reasonable assumption.

### 2.3 Reweighting – Introducing the Macro Shock at the Micro Level

To perform the analysis using EUROMOD, we reproduce the 2020 macroeconomic scenarios previously described in the EUROMOD microdata, based on EU-SILC. To do this, we rely on a reweighting approach, using it to translate the changes in several aggregate variables in the macroeconomic scenarios into changes at the microeconomic level. A similar approach has been used by, for instance, Dolls et al. (2019) to estimate the future impact of demographic change on the income distribution in Europe.

The key steps followed in our approach can be described as follows. First, we derive a set of key targets from the macroeconomic scenarios to be used as a basis for the reweighting. The key targets are total compensation of employees, total compensation of self-employed and total employment. It should be noted that targets are defined in terms of percentage changes compared to their value in 2017, i.e., the year of the underlying SILC data (for the detailed "shocks"- see Table A1 in the Appendix). Assuming that changes in employment translate one to one into unemployment changes (i.e., activity is held constant), we also derive an unemployment variation target. In this way, we derive the "shocks", allowing to reproduce in the underlying microdata the three different scenarios of 2020: (i) a 2020 without COVID-19 pandemic; (ii) a 2020 with COVID-19 and discretionary policy measures; and (iii) a 2020 with COVID-19 and without discretionary policy measures. We then introduce these shocks into the microdata, following the reweighting approach proposed by Pacifico (2014), which is formally described in Annex 5.

Intuitively, shocks are introduced in EUROMOD according to Table 1. The total employment target is met by adjusting the weight of people either declaring to be in work or reporting positive earnings (or earnings bigger than unemployment benefits). The double conditions on labour status and earnings derive from the construction of EU-SILC, which records benefits and earnings over an income reference period (usually the year previous to the interview) and other variables at the time of the interview. Similarly, the unemployment target is met by adjusting the weights of people in unemployment either with respect to the self-declared status or because in receipt of unemployment benefits.[13]

[12] https://euromod-web.jrc.ec.europa.eu/using-euromod/country-reports

[13] Since coverage rates of unemployment benefits can vary substantially across groups, having detailed information of a certain shock would increase the precision of our forecast of the unemployment expenditures. We account for coverage of unemployed, by not only adjusting the number of unemployed, but also the number of unemployment benefit receivers. Our approach assumes new unemployment to be similar to the unemployment observed, given their characteristics (unemployment duration, benefit amount…), which is of course a strong assumption.

**Table 1** Shock translation into EUROMOD

| | Information | EUROMOD | Shock/constant |
|---|---|---|---|
| Labour market structure | Employment increase (farmer, self-employed, employees) | les = 1,2,3 | shock |
| | Employment income receivers | yem > 0 & yem > ils_b1_bun | shock |
| | Unemployment | les = 5 | shock |
| | Unemployment benefit receivers | ils_b1_bun>0 | shock |
| | Pensioners | les = 4 | constant |
| | Students | les = 6 | constant |
| | Inactive | les = 7 | constant |
| | Sick or disabled | les = 8 | constant |
| | Other labour market status | les = 9 | constant |
| | Wage employed | yem | shock |
| | Earnings self employed | yse | shock |
| Demographic characteristics | Gender | dgn | constant |
| | Population group (0–15) | dag | constant |
| | Population group (16–40) | dag | constant |
| | Population group (41–65) | dag | constant |
| | Population group (>65) | dag | constant |

In two scenarios, the "No-COVID-19" and the "COVID-19 including discretionary policy changes", we additionally adjust weights such that the total wages of employees and earnings of self-employed match the respective targets in terms of compensation of employees and self-employed. The compensation measures account for wage compensation schemes in the scenario COVID-19 with discretionary policy measures. It should be noted that targets in terms of wage compensation are taken directly form the EC Economic Forecast for these two scenarios, while this is not the case for the scenario on COVID-19 without policy changes. For this reason, we opted for not imposing any additional condition on earnings in this scenario and let them vary unconstrained following the targeted employment changes.

We additionally ensure that the population structure stays constant by controlling for several age groups (0–15, 16–40, 41–65 years, as well as 65+) and labour market status, as well as for gender shares.

Our approach hence allows us to generate new datasets that reflect the macroeconomic conditions of the three scenarios of interest in terms of employment, unemployment and wages, including wage compensation schemes. Individual unemployment benefits as well as personal income taxes, social insurance contributions and other benefits from the reweighted simulation of EUROMOD are then aggregated at the country level to analyse the impact of the changes on the labour market (unemployment, wage loss) on households' income.

## 3 Main Findings

In this section, we present the main results of our analysis on the impact of the COVID-19 crisis, obtained by comparing the two COVID-19 scenarios, with and without discretionary policy measures, with the "No-COVID-19" scenario (baseline). Section 3.1 contains an analysis on EU-level, excluding Romania due to data limitations.[14] Section 3.2 presents

[14] The analysis excludes Romania because of the significant underreporting of unemployment benefits in the EUROMOD input datasets.

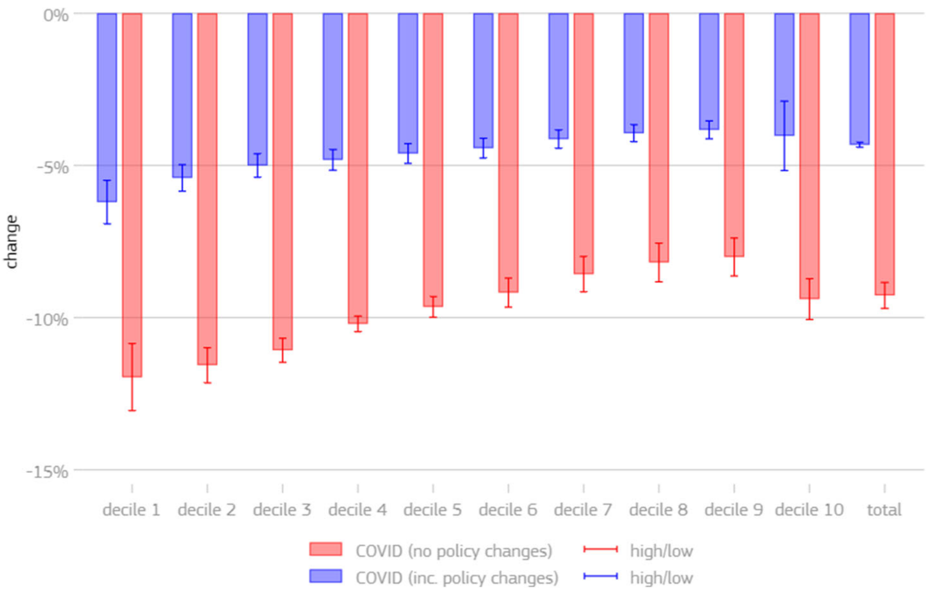

**Fig. 4** Impact of COVID-19 on equivalised disposable income in the EU, difference compared to no-COVID-19 scenario. Note: The impact of COVID-19 concerns 2020 and is estimated using equivalised disposable income by income decile. Household ranking is based on the income distribution of each scenario (No COVID-19, COVID-19 (no policy-changes) and COVID-19 (including policy-changes)). Results for each decile are weighted (population) average of country results. Source: own calculations using EUROMOD I2.0+

country-specific results. Here we consider only the medium scenario for the "COVID-19 no policy changes" scenario.[15]

### 3.1 Impact of COVID-19 on Household Income, Inequality and Poverty at the EU-Level

Figure 4 provides results on the impact of COVID-19 on average equivalised disposable income by income decile. Deciles are calculated using the income distribution of each scenario: No-COVID, COVID-19 (including policy changes) and COVID-19 (no policy changes). Results are shown for the two COVID-19 scenarios as the percentage change compared to the "No COVID-19" scenario. On average, compared to a hypothetical 2020 scenario without COVID-19 pandemic, household income would fall by −9.3% due to the impact of COVID-19 without fiscal policy measures, while policy intervention reduces this impact to −4.3%. In absence of fiscal policy responses, the COVID-19 pandemic would have a clear regressive effect on households´ income.

The reweighting procedure employed has a stochastic component which could affect the outcome of the reweighting algorithm. Therefore, we report the confidence intervals of our results by using a bootstrapping method (see Annex 7 for an in-depth discussion).

The first three lowest income deciles would experience a fall in equivalised disposable income oscillating between 12.0% and 11.1%. The fall in income for the three highest income deciles would represent a substantially lower decrease as experienced by the bottom income

[15] The detailed analysis of the low and high scenarios can be found in the Annex.

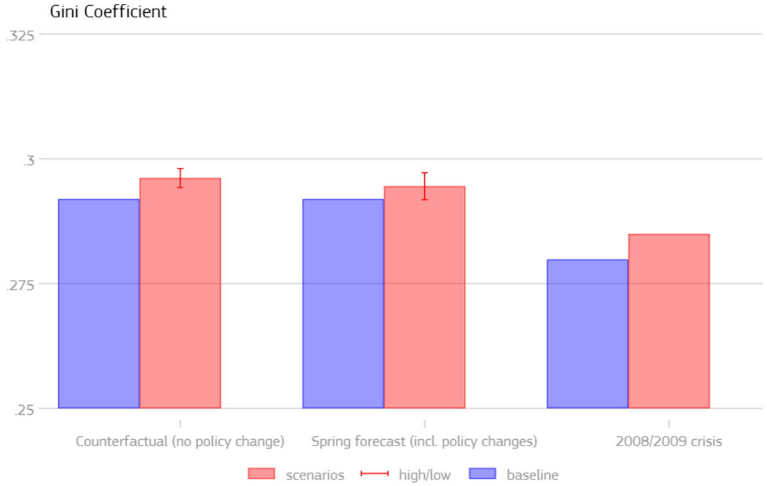

**Fig. 5** Impact of COVID-19 on income inequality in the EU. Note: The impact of COVID-19 concerns the year 2020. The baseline level of the Gini index is the weighted (population) average for 2020. The impact of the 2008–2009 crisis compares equivalised disposable income between these two years (EUROMOD baseline). Source: own calculations using EUROMOD I2.0 +

deciles, between −8.0% and − 9.4%. The COVID-19 pandemic would therefore affect households disproportionally, hitting lower income households the most.

Looking at the COVID-19 with policy change scenario, we can see that the fiscal measures taken by governments lead to a reduction of the regressive effect, resulting in a more homogeneous impact of about −6.2% to −3.8% all along the income distribution. This highlights that policy measures taken by the governments are likely to be effective at reducing both the size and the regressivity of the COVID-19 pandemic. It should be noted that we find a substantial cushioning effect on the richest decile, although our methodology leads to a substantial higher uncertainty in this decile.

Figure 5 provides a synthetic view on the impact of the COVID-19 crisis on income inequality by reporting the Gini index obtained according to the simulated scenarios and comparing these results with the impact of the 2008–2009 financial crisis. This figure highlights a number of important results. First, in absence of policy responses, the COVID-19 pandemic would trigger an increase in inequality, as measured by the Gini index, by 0.004. Policy measures, however, are able to counteract the inequality increasing effect of the COVID-19 pandemic slightly, as inequality in the scenario including policy measures would increase slightly less, by 0.003.[16] It is worthwhile mentioning that the results for the scenario with policy measures show a higher uncertainty. The inequality increase is in line with what was observed following the 2008–2009 crisis, which led to an increase in income inequality by 0.005 in the EU in its first year.

Figure 6 provides evidence on the potential impact of COVID-19 on poverty, measured by the At risk of poverty (AROP) rate (using the 60% of median income as threshold). These results are obtained by anchoring the poverty line to its 2020 value in the “No-COVID-19” scenario. According to the results, the AROP rate would increase significantly due to the

[16] The results on the Gini coefficient are likely to be influenced by the high impact of policy changes on high income households. We can see a substantial higher uncertainty for the COVID-19 including policy changes scenario, which is driven by the high uncertainty in the highest income decile.

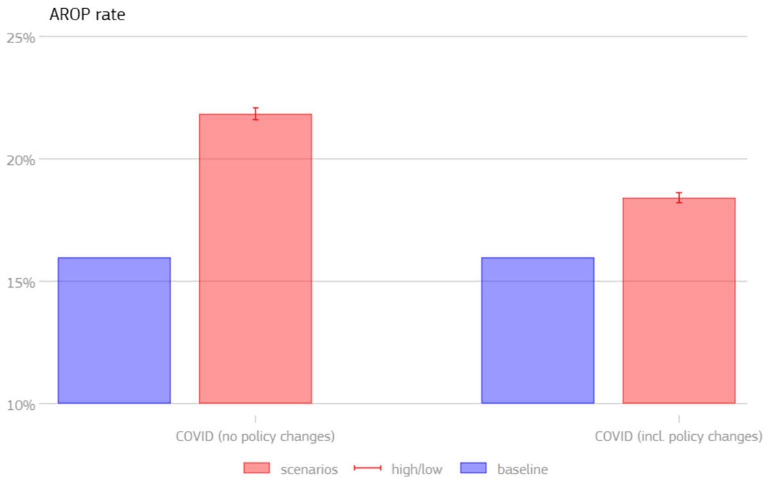

**Fig. 6** Impact of COVID-19 on poverty in the EU. Note: The impact of COVID-19 concerns 2020. The baseline level of the AROP indicator is the weighted (population) average for 2020. Source: own calculations using EUROMOD I2.0 +

COVID-19 pandemic, moving from 16.0% (2020 baseline) on average in the EU, up to 21.8% in the COVID-19 scenario without fiscal policy measures. When accounting for policy measures, this increase is less pronounced, from 16.0% to 18.4%.

Given the sheer size of the COVID-19 shock we might consider that the anchored poverty line may provide a more reliable assessment of the impact of the COVID-19 crisis on poverty.

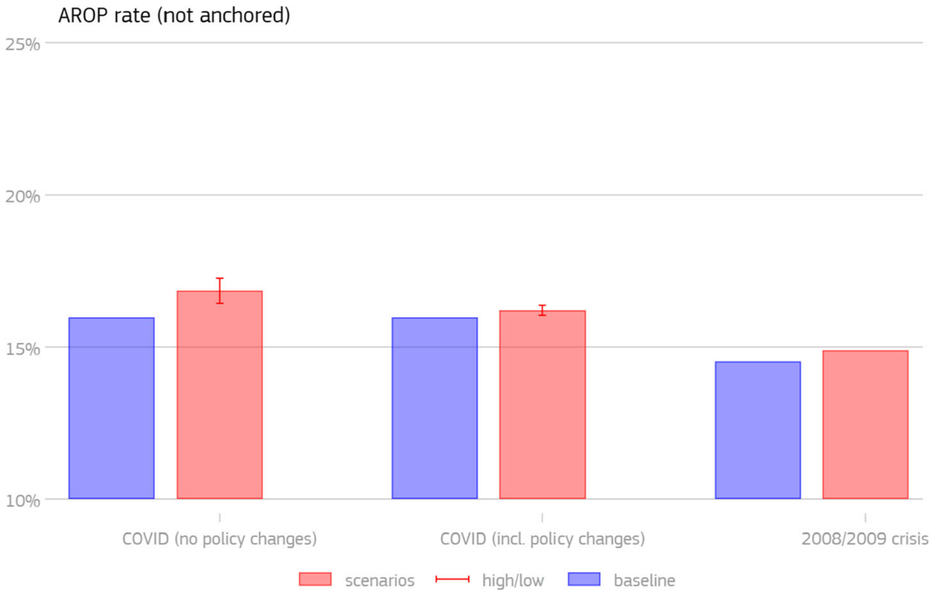

**Fig. 7** Impact of COVID-19 on poverty (non-anchored) in the EU. Note: The impact of COVID-19 concerns 2020. The baseline level of the AROP indicator is the weighted (population) average for 2020. The impact of the 2008–2009 crisis compares AROP indicator between these two years (EUROMOD baseline). Source: own calculations using EUROMOD I2.0+

Figure 7 provides evidence on the potential impact of COVID-19 on poverty, measured by the AROP rate (using the 60% of median income as threshold), when we do not anchor the poverty line to the No-COVID level. Again, the AROP rate would increase significantly due to the COVID-19 pandemic (+1 percentage point), but to a much smaller extent than in the event of fixed poverty thresholds. Policy measures can almost offset this increase in the AROP rate (+0.2 percentage points). When analysing these results, it should be kept in mind that the poverty line drops substantially in this analysis due to the income shock of the COVID-19 crisis.

By comparison, the 2008–2009 crisis implied increases in the AROP rate of about 0.4 percentage points. It follows that the current crisis is likely to have a similar social impact than the 2008–2009 crisis, at least in the short run.

### 3.2 Country-Specific Results

Additionally to the aggregate results at the EU-level, this subsection takes a closer look at the distributional impacts of the COVID-19 crisis, considering each of the EU Member States separately. Figure 8 shows the impact of the COVID-19 crisis on equivalised disposable income. Similar to Fig. 4, results are presented in terms of percentage difference with the "No-COVID-19" scenario. We consider first the medium policy scenarios (i.e., taking average values of the fiscal multipliers as indicated in Section 2.1). The income loss is expected to be especially high in countries such as Bulgaria, Estonia, Spain, Hungary, Ireland, Lithuania, Latvia, Poland and Slovakia. Still, policy measures can offset this income loss considerably. In countries such as Austria, Bulgaria, Germany, Estonia, Hungary, Latvia and Malta, the policy measures taken can reduce the income losses substantially. Despite the policy measures, we expect the biggest impact of the COVID-19 pandemic on equivalised disposable income in countries such as Bulgaria, Cyprus, Czechia, Spain, Croatia, Hungary, Lithuania and Poland.

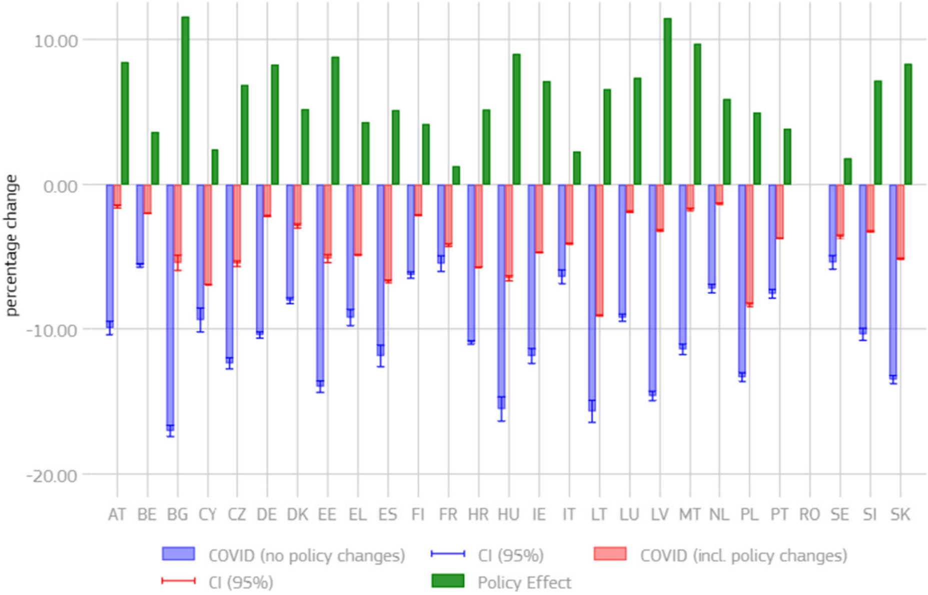

**Fig. 8** Impact of the COVID-19 crisis on equivalised disposable income in EU countries. Source: own calculations using EUROMOD I2.0+

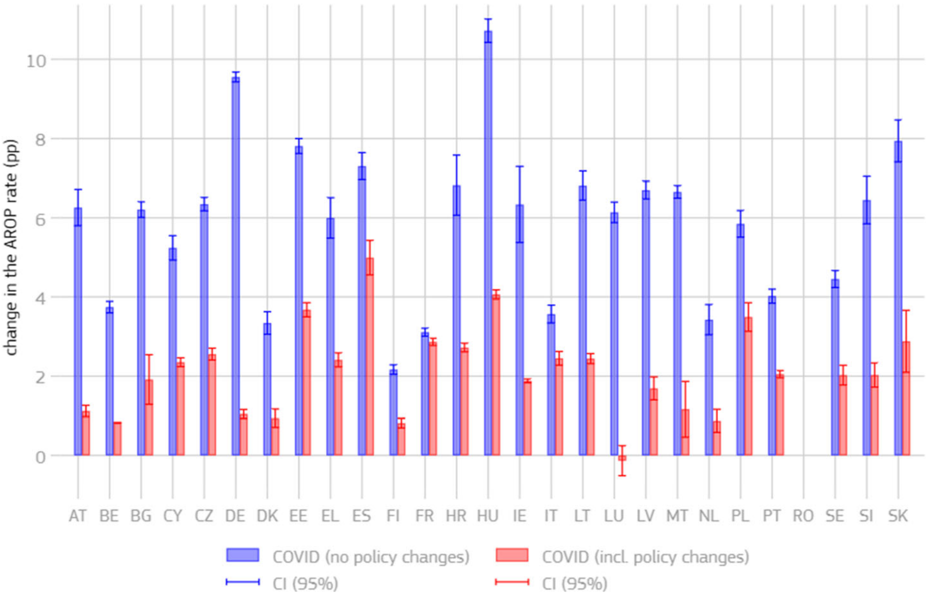

**Fig. 9** Impact of the COVID-19 crisis on poverty (AROP rate) in EU countries. Source: own calculations using EUROMOD I2.0+

Figure 9 shows the impact of the COVID-19 crisis on poverty, measured by the AROP rate, where the poverty line is anchored to the value of the "No-COVID-19" scenario (baseline). The data are reported as difference in poverty rate in each COVID-19 scenario compared to the "No-COVID-19" scenario. Not surprisingly, the AROP rate jumps substantially in the counterfactual scenario, where no policy measures are taken. This is due to a substantial decrease in household income following the increase in unemployment that differs across countries. Substantial increases of over 5 pp. in the AROP would take place in many Member States in absence of policy interventions. Especially high poverty rates would be observed in the hypothetical scenario with no policy intervention (blue bars) in Germany, Estonia, Spain, Hungary and Slovakia. Strong increases can be also seen in Croatia and Lithuania. When we consider the policy measures taken by the governments, we can see that the impact of COVID-19 on the AROP rate can be alleviated in many countries and in some almost offset, particularly in Belgium, Germany, Denmark, Finland, Luxemburg and the Netherlands.[17]

Figure 10 shows the impact of the COVID-19 crisis on inequality, measured by the differences in the Gini index between each COVID-19 scenario and the scenario without COVID-19. In many countries, policy measures are able to partly offset the inequality-increasing pattern of the COVID-19 pandemic, but there are some exceptions. For example, in Bulgaria, Finland, France and Poland policy measures may significantly increase the Gini index. It is also worth noting that in some countries, the confidence intervals are quite large.

[17] If we consider the non-anchored poverty rate in Figure A6 in the Annex, we can see that the impact on the poverty rate is not as strong as in the case of an anchored poverty rate. The results are driven by a substantial drop of the poverty line in both, the COVID-19 scenario with and without policy measures compared to baseline. As Figure 8 already showed, both shock scenarios lead to a severe reduction in equivalised disposable income, shifting the income distribution and therefore the poverty line to the left. Keeping this in mind it is not surprising that in the case of the shock scenarios, the non-anchored poverty reacts less to the COVID-19 shocks than the anchored one.

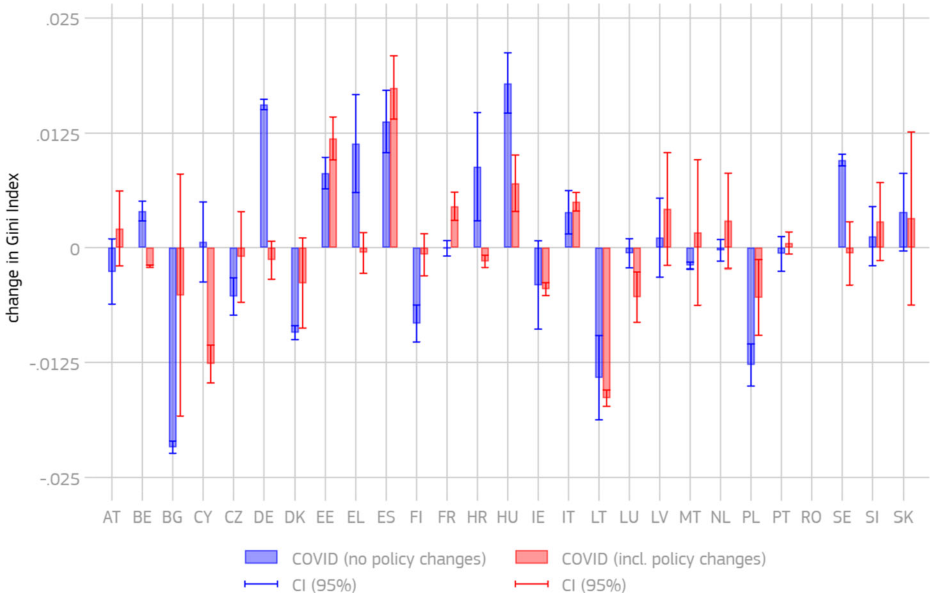

**Fig. 10** Impact of the COVID-19 crisis on inequality (Gini index) in EU countries. Source: own calculations using EUROMOD I2.0+

The above results are driven by the impact of the COVID-19 shock on household income. While in the scenario of COVID-19 without policy measures all people losing their job are sent into unemployment, in the case of the COVID-19 scenario with policy measures, the government tries to counteract the loss in household income by policies such as wage compensation schemes. To get a clearer picture on the impact on the income distribution, we additionally take a closer look at the impact on household income by income decile in each country, see Figs. A7 in the Annex. We can see that in most of the countries, policy measures can offset the regressive impact of the COVID-19 pandemic, although it should be noted that there is substantial uncertainty in our results when it comes to the impact on the top and the bottom part of the income distribution. We must acknowledge that our approach faces some limitations. Strong assumptions regarding the way the macroeconomic shock related to COVID-19 is translated at the micro-level and in the determination of the baseline scenario are needed. We have conducted a number of robustness checks in order to address these limitations. The detailed checks can be found in Annex 6 and 7.

As a robustness check we also consider the low and high scenarios (corresponding to low and high fiscal multipliers) in our analysis. Fig. A3 in the Annex confirms the robustness of the main findings in the previous section across the assumptions on fiscal multipliers, although in some cases the impact of the crisis would be rather pronounced under the high scenarios compared to the medium one.

## 4 Conclusion

The consequences of the COVID-19 crisis on households′ income, although still unknown with precision, raise serious concerns. In this paper, we provide an assessment of the potential impact of the fiscal policy measures adopted in the wake of the COVID-19 crisis on household income, poverty and inequality in the EU in 2020. In particular, we used the EUROMOD microsimulation model for the EU to compute the impact of aggregate GDP and employment changes on households' incomes.

We built our results around three macroeconomic scenarios. The first scenario corresponds to the EC Spring 2020 Economic Forecast, including the estimated impacts of the COVID-19 crisis, such as the shutdown of major parts of the economy as well as the policy measures taken by Member States to counteract the strong impact of the pandemic. The second scenario is a no policy-change scenario, excluding discretionary fiscal policy measures. This hypothetical scenario is built to gauge the effect of the fiscal policy measures taken by EU countries. Both scenarios are evaluated in terms of differences with the economy in absence of COVID-19.

Next, we reweighted the underlying EUROMOD survey microdata from the European Union Statistics on Income and Living Conditions (EU-SILC) to mimic the aggregate employment figures in each scenario. In particular, we make use of the information on employment and unemployment changes in the forecasts, as well as changes in the total wage compensation for employees and the self-employed to simulate the impact of COVID on (un)employment, as well as on wage compensations.

Our analysis suggests that over the course of 2020, on average, equivalised disposable income in the EU would fall by −9.3% due to the COVID-19 crisis without discretionary fiscal policy measures, and by −4.3% with policy intervention, pointing to a significant cushioning effect of these measures protecting households against income losses. Furthermore, our results confirm that the impact of the COVID-19 crisis is likely to be highly regressive, with an increase in the number of poor households. However, discretionary policy measures are expected to partly contain the regressive effects of the recession. Policy interventions are therefore instrumental in cushioning against the impact of the crisis, especially on poverty.

In addition to the aggregate results at EU-level, we presented results for each of the EU Member States. Despite some exceptions, Member States' policy measures prove their worth in limiting poverty and inequality at the country level. Poverty, as measured by the At risk of poverty rate (AROP) rate, would increase significantly in absence of fiscal policy measures. However, when accounting for the policy measures taken by the governments, we observe that the impact of COVID-19 on the AROP rate can be alleviated in many countries and in some almost offset. In many countries, policy measures are also able to offset the inequality-increasing pattern of the COVID-19 pandemic.

**Supplementary Information** The online version contains supplementary material available at https://doi.org/10.1007/s10888-021-09485-8.

**Acknowledgements** We are thankful for comments on preliminary results received from Olivier Bontout, Peter Benczur, Daniel Daco, Lucie Davoine, Giulio Paso and Sergio Torrejón Pérez. The views expressed in this paper should not be attributed to the European Commission.

**Open Access** This article is licensed under a Creative Commons Attribution 4.0 International License, which permits use, sharing, adaptation, distribution and reproduction in any medium or format, as long as you give appropriate credit to the original author(s) and the source, provide a link to the Creative Commons licence, and indicate if changes were made. The images or other third party material in this article are included in the article's Creative Commons licence, unless indicated otherwise in a credit line to the material. If material is not included in the article's Creative Commons licence and your intended use is not permitted by statutory regulation or exceeds the permitted use, you will need to obtain permission directly from the copyright holder. To view a copy of this licence, visit http://creativecommons.org/licenses/by/4.0/.

## Supplementary Information

ESM 1 (PDF 984 kb)

ESM 2 (XLSX 143 kb)
