# Supplementary material for: The impact of COVID-19 on households´ income in the EU
Source: J Econ Inequal. 2021 Jun 1;19(3):413–31. doi: 10.1007/s10888-021-09485-8 (PMC8167381; doi:10.1007/s10888-021-09485-8)
Supplement: Supplementary file 1 — (PDF 984 kb) [file 10888_2021_9485_MOESM1_ESM.pdf]

Annex 1: Changes in COVID-19 scenario

Table A1: Shock scenario by country (changes in %, 2017-2020)

**Baseline (AF)**	**AT**	**BE**	**BG**	**CY**	**CZ**	**DE**	**DK**	**EE**	**EL**
Compensation of Employees, Euros	12.7%	9.1%	29.0%	17.2%	25.9%	11.9%	11.5%	26.9%	14.4%
Compensation of Self Employed, Euros	4.4%	9.9%	20.2%	14.3%	21.7%	5.6%	7.4%	34.9%	-1.5%
Total Employment Growth, Persons	3.5%	3.5%	0.5%	9.0%	3.1%	2.2%	3.9%	2.0%	6.2%
**Baseline (AF)**	**ES**	**FI**	**FR**	**HR**	**HU**	**IE**	**IT**	**LT**	**LU**
Compensation of Employees, Euros	12.9%	11.4%	6.1%	13.3%	17.8%	18.4%	7.1%	23.0%	21.7%
Compensation of Self Employed, Euros	6.8%	10.4%	4.7%	6.7%	29.0%	14.3%	4.6%	17.3%	18.8%
Total Employment Growth, Persons	5.4%	4.1%	2.7%	4.4%	4.5%	7.4%	1.5%	1.7%	11.2%
**Baseline (AF)**	**LV**	**MT**	**NL**	**PL**	**PT**	**RO**	**SE**	**SI**	**SK**
Compensation of Employees, Euros	23.7%	21.4%	13.6%	25.8%	13.6%	43.5%	0.1%	20.0%	23.5%
Compensation of Self Employed, Euros	12.1%	25.6%	10.3%	25.1%	9.4%	24.3%	-6.8%	17.6%	19.3%
Total Employment Growth, Persons	1.2%	13.0%	5.0%	0.3%	3.9%	0.5%	2.1%	7.1%	3.1%
**COVID with Policy (SF)**	**AT**	**BE**	**BG**	**CY**	**CZ**	**DE**	**DK**	**EE**	**EL**
Compensation of Employees, Euros	8.8%	4.2%	20.3%	2.6%	15.3%	7.7%	5.1%	16.3%	1.4%
Compensation of Self Employed, Euros	3.8%	3.8%	10.0%	1.0%	10.6%	-0.2%	0.3%	24.5%	-6.9%
Total Employment Growth, Persons	1.5%	1.9%	-2.2%	4.6%	-1.1%	1.4%	0.9%	-3.3%	0.0%
**COVID with Policy (SF)**	**ES**	**FI**	**FR**	**HR**	**HU**	**IE**	**IT**	**LT**	**LU**
Compensation of Employees, Euros	0.3%	5.5%	0.4%	2.2%	6.6%	8.8%	-3.0%	7.3%	16.0%
Compensation of Self Employed, Euros	-6.7%	10.4%	1.1%	1.5%	23.2%	-0.6%	-5.9%	-0.6%	13.1%
Total Employment Growth, Persons	-4.7%	0.9%	-7.1%	-0.7%	0.1%	3.4%	-6.2%	-1.7%	8.4%
**COVID with Policy (SF)**	**LV**	**MT**	**NL**	**PL**	**PT**	**RO**	**SE**	**SI**	**SK**
Compensation of Employees, Euros	18.3%	17.4%	9.6%	10.2%	6.6%	20.8%	-6.2%	13.0%	13.8%
Compensation of Self Employed, Euros	8.0%	29.5%	6.3%	5.3%	-3.8%	6.2%	-15.2%	15.1%	10.6%
Total Employment Growth, Persons	-1.2%	9.9%	1.8%	-4.2%	-0.4%	-2.3%	-0.4%	2.8%	-0.2%
**No Policy**	**AT**	**BE**	**BG**	**CY**	**CZ**	**DE**	**DK**	**EE**	**EL**
Compensation of Employees, Euros	na	na	na	na	na	na	na	na	na
Compensation of Self Employed, Euros	na	na	na	na	na	na	na	na	na
Total Employment Growth, Persons	-6.7%	-5.5%	-8.7%	-5.0%	-8.4%	-8.2%	-6.9%	-7.6%	-11.7%

No Policy	ES	FI	FR	HR	HU	IE	IT	LT	LU
Compensation of Employees, Euros	na	na	na	na	na	na	na	na	na
Compensation of Self Employed, Euros	na	na	na	na	na	na	na	na	na
Total Employment Growth, Persons	-7.9%	-3.8%	-7.2%	-10.0%	-8.1%	-5.6%	-9.5%	-10.9%	-4.6%

No Policy)	LV	MT	NL	PL	PT	RO	SE	SI	SK
Compensation of Employees, Euros	na	na	na	na	na	na	na	na	na
Compensation of Self Employed, Euros	na	na	na	na	na	na	na	na	na
Total Employment Growth, Persons	-7.8%	1.6%	-5.2%	-6.0%	-4.7%	-6.0%	-5.1%	-5.3%	-6.4%

Source: European Commission Spring Forecast 2020 and Autumn Forecast 2019, own calculation. Note: For France, we had to assume a wage compensation growth in the COVID with policy scenario due to data issues in the Spring Forecast. We estimated a pattern in line with the employment growth - wage compensation growth relation as in other countries.

Annex 2: Employment changes in no policy-change scenarios

Table A2: Employment changes in no policy-change scenarios

Country	EMPLOYMENT IMPACT (% change)			
	**EC Spring 2020 Economic Forecast**	**LOW**	**MEDIUM**	**HIGH**
AT - Austria	-1.4	-7.17	-8.66	-10.45
BE - Belgium	-1.0	-7.03	-7.33	-7.69
BG - Bulgaria	-2.5	-8.11	-8.90	-9.84
CY – Cyprus	-2.5	-8.40	-9.36	-10.51
CZ - Czechia	-3.1	-8.16	-9.57	-11.24
DE - Germany	-0.9	-8.59	-10.20	-12.10
DK - Denmark	-1.6	-7.33	-8.42	-9.73
EE - Estonia	-5.7	-8.67	-9.82	-11.18
EL- Greece	-3.7	-11.32	-12.65	-14.23
ES - Spain	-8.7	-9.99	-10.77	-11.69
FI - Finland	-2.5	-6.23	-6.61	-7.07
FR - France	-9.1	-8.22	-8.59	-9.04
HR - Croatia	-3.9	-10.51	-11.77	-13.27
HU - Hungary	-3.8	-9.65	-11.57	-13.85
IE - Ireland	-2.5	-8.90	-9.37	-9.93
IT - Italy	-7.5	-10.27	-10.61	-11.01
LT - Lithuania	-3.5	-10.42	-12.36	-14.67
LU - Luxembourg	0.9	-6.73	-7.91	-9.30
LV - Latvia	-2.6	-8.30	-9.21	-10.30
MT - Malta	-1.8	-5.46	-6.21	-7.10
NL - Netherlands	-2.4	-7.74	-8.58	-9.59
PL - Poland	-4.5	-5.40	-6.40	-7.59
PT - Portugal	-3.4	-6.66	-7.01	-7.44
RO - Romania	-2.5	-5.57	-6.05	-6.62
SE - Sweden	-2.5	-6.70	-7.15	-7.70
SI - Slovenia	-2.7	-7.70	-8.82	-10.15
SK - Slovakia	-3.4	-8.14	-9.11	-10.28
**AVERAGE**	**-4.4**	**-8.04**	**-8.97**	**-10.09**

Source: European Commission Spring Forecast 2020, own calculations.

While the employment impact in the COVID no policy-change scenario is logically more severe than in the EC Spring 2020 Economic Forecast, which accounts for the mitigating

impact of compensation measures such as Short-Time Work schemes, it is in line with the historically observed relationship between GDP growth and employment changes. This is illustrated in Figure A1, which plots the relationships observed for each of the EU Member States since 2001 (as coloured dots). Moreover, it shows those for the EC Spring 2020 Economic Forecast (light blue circles) and those for the no policy-change scenario (black circles).

Figure A1: GDP growth vs % change in employment

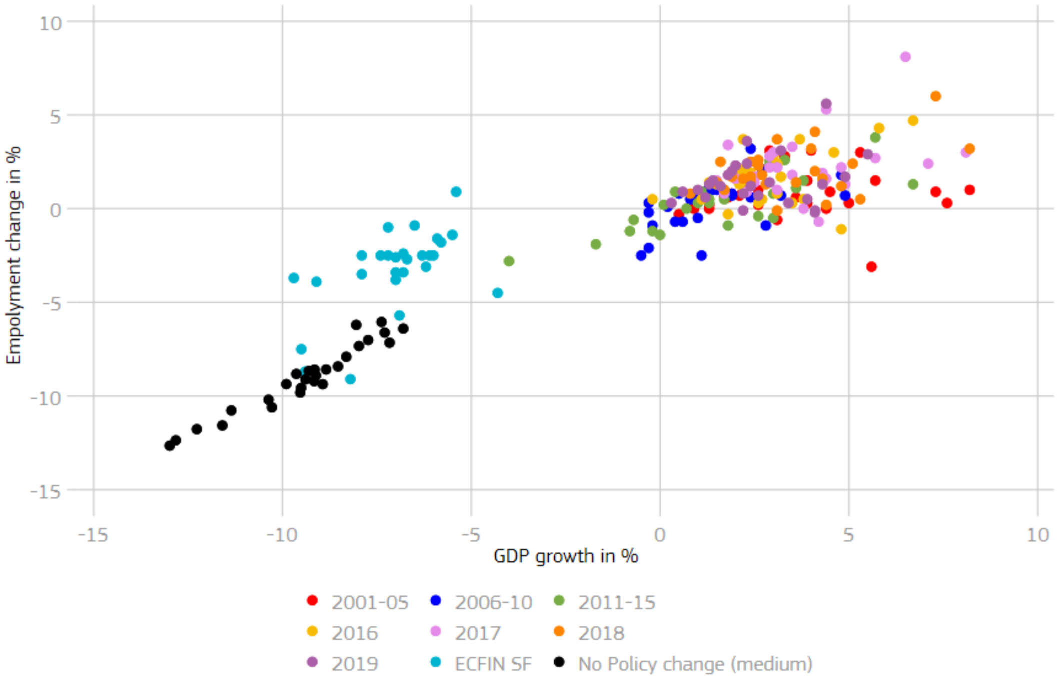

Annex 3: Shock scenarios and the impact on the income distribution

Figure A2: Shock scenarios and the impact on the income distribution

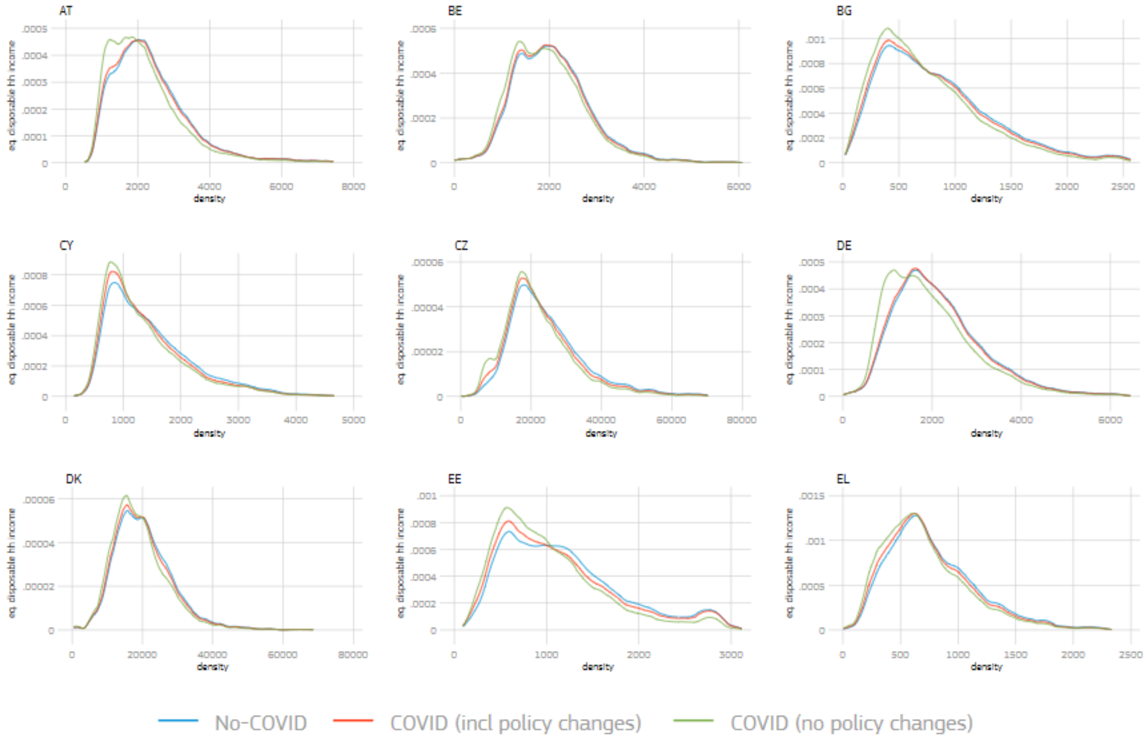

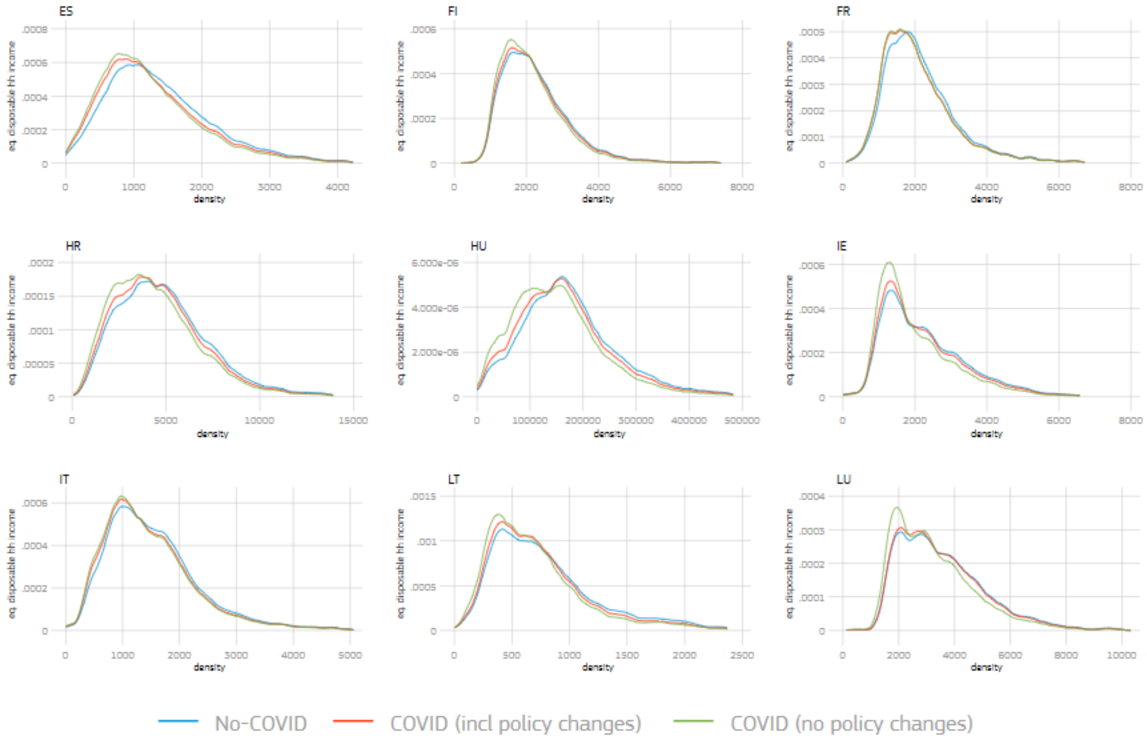

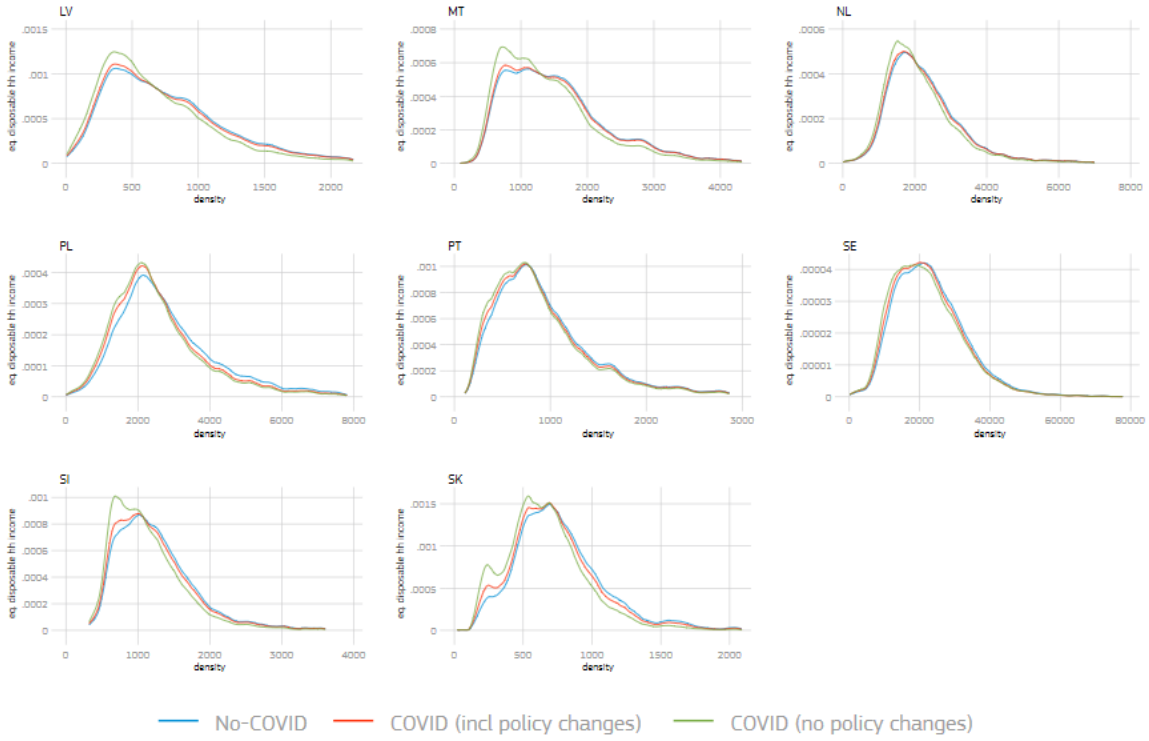

Source: own calculations using EUROMOD I2.0+.

Annex 4: Notes on the EUROMOD methodology

As any simulation exercise, the approach presented in this paper is subject to some caveats that are worthwhile mentioning:

- We use the policy system of 2019, with underlying EU-SILC data from 2017 (income year 2016).
- The concept of expenditures and revenues of EUROMOD might be different to standard National Accounting concepts. Therefore, we stick to reporting percentage changes for our forecasts.
- Our method generates a new population (given the expected employment changes) due to reweighting. Therefore, we are not able to follow the same person in both populations. We have to change the income deciles accordingly when comparing scenarios. This approach is in line with comparing changes over years, which has of course some limitations.
- One assumption that we have to use, is that all unemployed have similar characteristics as the current unemployment pool. We cannot take into account that new unemployed might differ from the pool of currently unemployed.

Moreover, from a methodological point of view it is worth noting that:

- While EUROMOD is based on household data, some benefits and taxes (especially on the firm side) are not included in the data. Additionally, indirect taxes and in-kind benefits cannot be taken into account on the household side.
- Although the proposed methodology captures the heterogeneity in unemployed characteristics, an (un)employment shock originated by aggregated demand will generate the same pool of unemployed as an aggregated supply shock of the same

size. A possible way to overcome this limitation is to use additional information on the type of shock in the selection process. For example, different types of shock may affect workers asymmetrically with respect to the skill level or industries. We can simulate this with our approach, but detailed information on the sector/skill shocks would be needed for this purpose.

- Finally, it should be noted that reweighting may perform worse compared to a transition approach in times of rapid economic changes, e.g., if individuals entering in unemployment have characteristics completely different from the characteristics of the unemployed observed in the base year.

Annex 5: Reweighting procedure

Formally, let us consider a survey of N individuals and K individual-level variables, such as income, gender, working status and age: $x_{i,} = (x_{i,1}, x_{i,2}, \dots, x_{i,K})$. The survey weight is defined as a vector $s = (s_1, s_2, \dots, s_N)$ of all individual weights. The estimated 1 × K vector of survey totals is given by:

$$t = \sum_{i=1}^{N} s_i * x_i$$

Since we are interested in introducing a shock into our data, we are particularly interested in changes to specific group totals (while other population characteristics might stay the same) to get a realistic dataset for 2020 that includes our shock. It is possible to compute a new vector of survey weights $w = (w_1, w_2, \dots, w_N)$ that is as close as possible to the original weights and that respects the following calibrating conditions,

$$t_{new} = \sum_{i=1}^{N} w_i * x_i$$

where t_{new} is the 1 × K vector of projected total values including the shock. Let us assume that the distance between the original and the new weights is following a distance function $g(s_i, w_i)$, then the new weights can be obtained by minimising a Lagrangian function with respect to the new weights:

$$L = \sum_{i=1}^{N} g(s_i, w_i) + \sum_{k=1}^{K} \lambda_k * \left(t_k - \sum_{i=1}^{N} w_i * x_{i,k} \right)$$

where $\lambda = (\lambda_1, \lambda_2, \dots, \lambda_K)$ are the Lagrange multipliers. The solution of the minimisation problem depends on the properties of the chosen distance function. We use the distance

function proposed by Deville and Särndal (1992) which keeps the calibrated weights within a known range set.[19]

Annex 6: Robustness of the results regarding Macro scenarios

We consider three possible macro scenarios for the quarterly evolution of the multipliers described in Section 2.1: a low, a medium and a high scenario. The multipliers considered in each quarter in each of the three scenarios are presented in Figure 2. Figure A3 highlights the variation of the country-specific results, in those different scenarios.

Figure A3: Impact of the macro scenarios on country-specific results

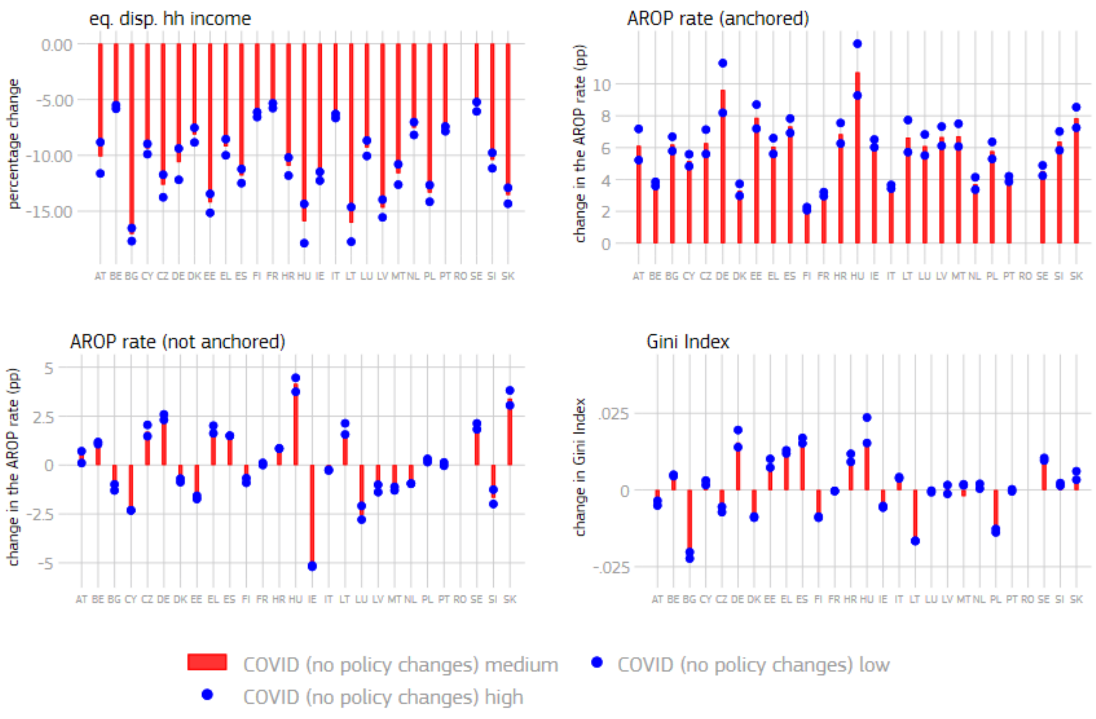

Source: own calculations using EUROMOD I2.0+.

[19] For more information, see Pacifico (2014).

Annex 7: Robustness of the country-specific results

First, we use the policy system of 2019, with underlying EU-SILC data from 2017 (income year 2016) which are uprated to 2019 prices. Second, the concept of expenditures and revenues of EUROMOD might be different to standard national accounting concepts that are used on macro level. To overcome both problems, we stick to reporting and using percentage changes when introducing the shock in the data.

Our method generates a new population (given the expected labour market and demographic changes) due to reweighting. Therefore, we are not able to follow the same person in both populations. We have to change the income deciles accordingly when comparing scenarios. This approach is in line with comparing changes over years.

When we introduce the unemployment shock, we assume that all new unemployed have similar characteristics as the current unemployment pool. Hence, we cannot take into account the fact that the newly unemployed might differ from the pool of currently unemployed. It should also be noted that reweighting may perform worse compared to a transition approach in times of rapid economic changes, e.g., if individuals entering in unemployment have characteristics completely different from the characteristics of the unemployed observed in the base year.

Furthermore, we do not have information on which employees are especially hit by a wage loss (that also stems from short-time working schemes). By using a reweighting approach, the survey weights of employees with higher wages are shifted to employees with lower wages. This approach does not take into account any distributional pattern that wage loss could potentially have. Other studies show, that essential workers are often based in the lower income deciles, while home-office possibilities are typically more likely for high-income

earners, see for instance Galasso (2020) for an analysis specific to the COVID-19 pandemic on labour markets. Hence, there is evidence that people in the middle-upper part of the distribution are more likely to move to short-time work. Additionally, since we do not explicitly simulate compensation schemes but we rather reweight to take them into account, the potentially heterogeneous effect of these schemes across the income distribution (like e.g., upper limits in the wage compensation schemes, which lead to higher wage drops in the upper income distribution) are also not considered in our approach.

To ensure the robustness of our results, we introduce a bootstrapping procedure that allows the algorithm to be more flexible in the weight choice. In the baseline, the algorithm specifies the upper and lower bound of the ratio between the new and the original weight when the Deville and Sarndal's distance function is used. To ensure that the choice of the boundaries does not affect our solution, we do bootstrapping on randomly chosen bounds in the algorithm.[20] Therefore, we get a sequence of results that allow us to build an average effect with a standard error.

The bootstrapping procedure allows us to test the statistical significance of our results. The confidence intervals are represented in all the country-specific figures. We can see that these standard errors are especially high in countries such as Cyprus, Lithuania, Czechia and Hungary. Additionally, we can see that when looking at the distributional country-specific analysis, the uncertainty of the results is especially high in those countries and especially in the higher deciles. This is driven by the fact that the share of employees, who are those hit by the crisis (either by losing their job or going into wage compensation) is the highest in the

[20] In the baseline, the value default for the upper bound is 3 and for the lower bound 0.2.

upper deciles in most countries. This limitation must be taken into account when considering our results on the distributional effects of our different scenarios.

We must also keep in mind that our approach randomly reduces wages, although the COVID-19 shock might hit specific groups of workers (high-skilled vs. low-skilled, male vs. female, young vs. old), and sectors differently. Our simulations capture sector composition of the change in employment/unemployment when converting the GDP shock into employment shocks without considering the differential wage impact across sectors, see Section 2.

Using information available in the EU-SILC data on the sector of activity we can partially account for these effects. In such case the differential impact of the crisis by worker category would reflect *only* the sectoral differences in skill/gender/age composition of the workforce. This is consistent with Fana et al. (2020) who argue that the sectorial impact of the crisis affects substantially the distributional impact. The authors show that those sectors that were closed or only partly active have on average lower wages than those that were likely to be essential or where teleworking was possible. Consequently, the sectorial structure of the shock can potentially have an important impact on the income distribution that we do not account for.

Those patterns seem to be quite similar across countries, as highlighted in Table A3. Therefore, as an additional robustness check, we consider the impact on the wage loss (wage compensation) only in those sectors that are mostly affected by the COVID-19 pandemic. Following Fana et al. (2020) those are: Construction, Wholesale and retail, Hotels and restaurants and Transport and communication.

Figure A4 compares the results of our simulations on the impact of COVID-19 on households´ income in our main scenario (including policy measures) with a scenario where

the sector dimension is considered as described above. In some cases, the simulated change in households´ disposable income is larger or smaller than in our main results. However, these differences are arguably very small. For instance, the largest difference in results can be observed for Sweden and Malta indicating a larger (in absolute terms) decrease in households´ disposable income, which represents only a very small portion of the simulated fall in our main results. Results at decile level reveal the same type of results and leave the distributional pattern of COVID across countries broadly unchanged compared to our main results too.[21]

Figure A4: Accounting for sector specific shocks: differences in equivalised disposable income in benchmark results vs. results incorporating sector-specific impact of COVID-19

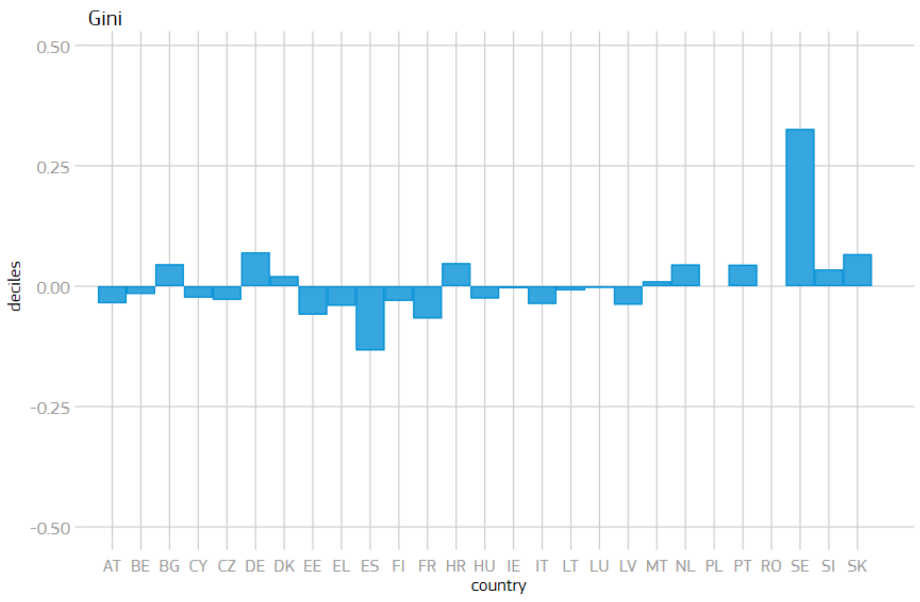

Source: own calculations using EUROMOD I2.0+.

Figure 12 highlights the impact of the sector allocation on the AROP rate as well as on the Gini index compared to our benchmark results incorporating the impact of policy change. Not surprisingly, the impact on both measures is quite similar in all countries, since both, the

[21] Only for the 1st and 10th decile results can vary more for some countries.

AROP and the Gini index are measures of income inequality. While in most countries, the sector specific shock would lead to a higher AROP rate and Gini coefficient, in some the opposite holds true.

Additionally to those robustness checks, Figure A3 highlights the impact of the assumptions underlying the macroeconomic scenario on which the counterfactual scenario in section 2.1 is constructed. We conclude that the country-specific impact can differ substantially depending on the choice of the multipliers when creating the counterfactual scenarios. Using multipliers to the higher end of the spectrum (as often found for recessions in narratively identified empirical models), for instance, would result in considerably more negative growth rates in the counterfactual scenario. Nonetheless, the physical lockdown of economies is likely to have temporarily limited marginal propensities to consume and, thus, the size of the multiplier for the fiscal impulses enacted in this period.

Figure A5: Accounting for sector specific shocks: differences in inequality and poverty in benchmark results vs. results incorporating the sector-specific impact of COVID-19

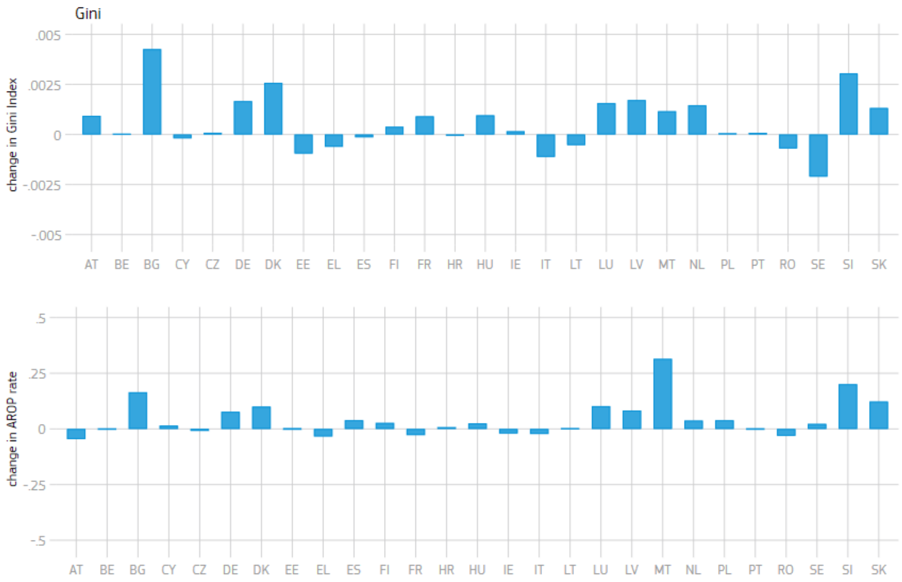

Source: own calculations using EUROMOD I2.0+.

Annex 8: Additional figures and tables

Table A3: Average wage percentile of jobs in each of the categories

	Essential	Teleworkable	Partly active	Mostly non-essential	Closed	All sectors
DE	46.1	67.6	36.8	55.3	27.5	50
FR	45.5	64.1	41.4	51.1	35.5	50
IT	53.6	72.5	38.2	45.8	25.6	50
ES	53.8	71.7	34.9	47.4	31.6	50
PL	45.6	69.8	36.0	50.4	33.6	50
NL	48.5	69.2	35.9	51.6	26.8	50
RO	50.6	66.7	39.3	50.3	27.0	50
CZ	53.5	67.2	36.5	47.1	29.4	50
SE	42.7	64.0	43.6	51.3	29.0	50
BE	46.4	67.1	36.4	51.0	30.1	50
HU	49.9	61.2	40.5	47.8	41.6	50
AT	48.2	66.6	38.0	56.0	25.0	50
GR	44.4	74.9	40.9	48.3	29.0	50
PT	44.3	74.2	48.5	33.2	36.7	50
BG	48.9	67.4	43.8	45.1	36.5	50
FI	41.4	69.4	40.5	56.7	29.7	50
SK	50.6	61.3	37.8	51.3	31.1	50
DK	46.6	71.5	37.9	51.1	23.9	50
IE	51.2	75.0	28.4	52.8	21.3	50
HR	53.5	68.1	36.5	45.3	31.0	50
LT	46.6	66.1	43.0	49.4	32.1	50
SI	48.7	69.8	42.8	42.9	33.4	50
LV	48.9	65.8	42.1	46.7	35.0	50
EE	47.8	61.7	43.0	52.9	30.9	50
CY	53.3	72.3	37.2	42.9	25.3	50
LU	44.0	61.9	29.5	53.1	24.5	50
MT	51.8	67.3	32.4	44.5	40.3	50

Source: Fana et al. (2020)

Figure A6: Impact of the COVID-19 crisis on AROP rate (non-anchored) in EU countries

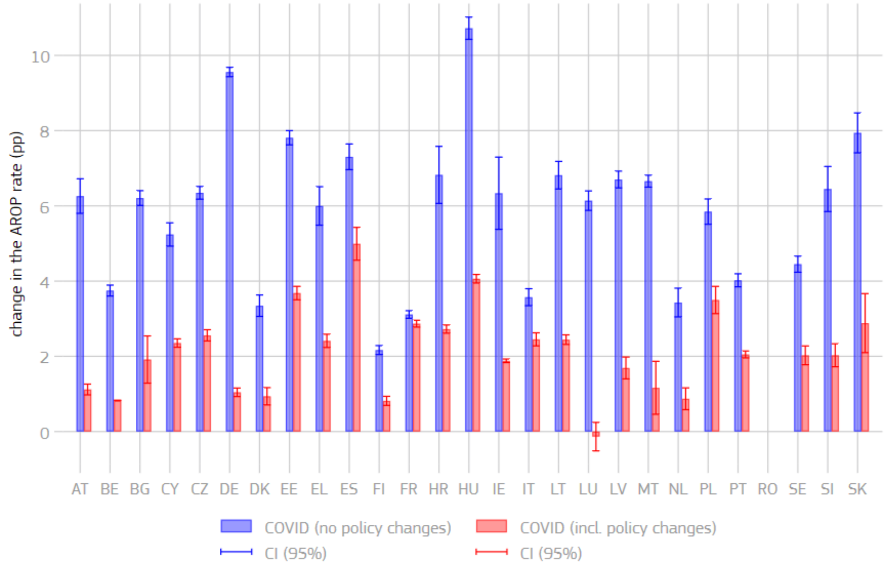

Source: own calculations using EUROMOD I2.0+.

Figure A7: Change (%) in equivalised disposable income by decile

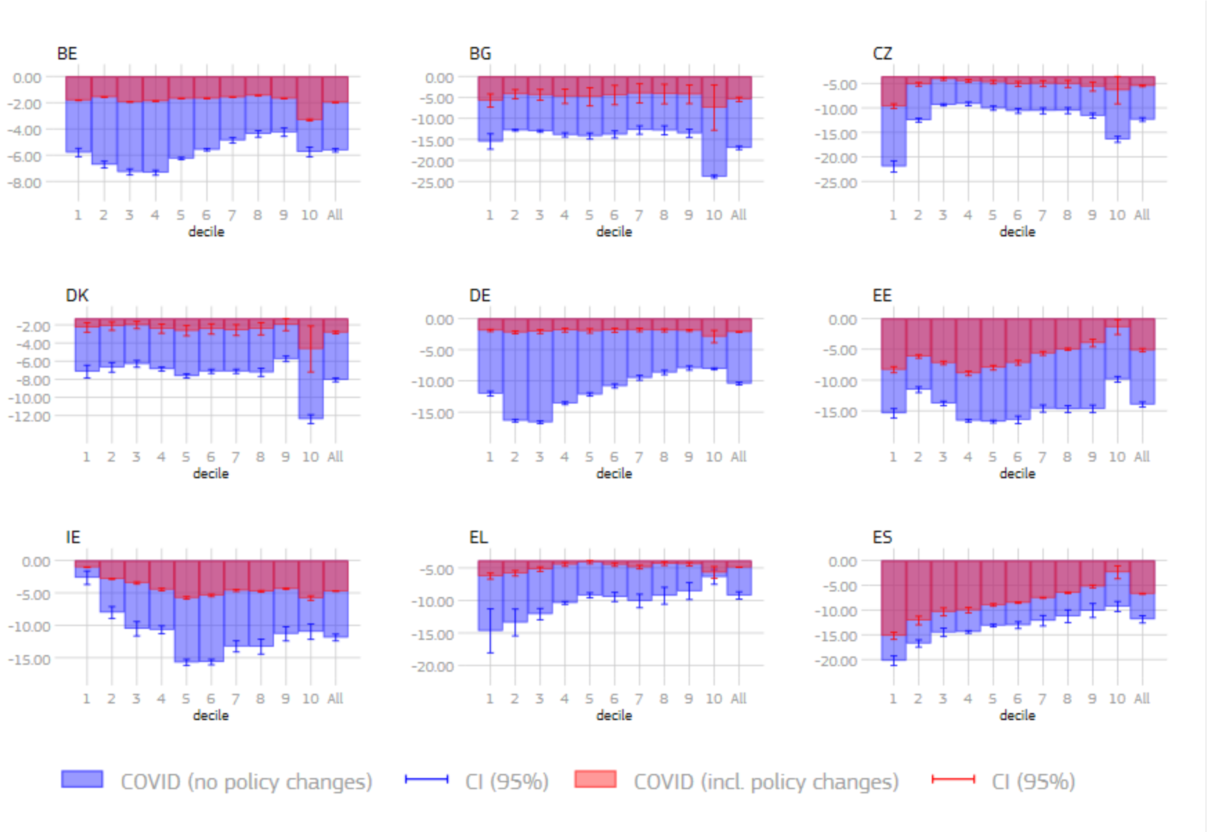

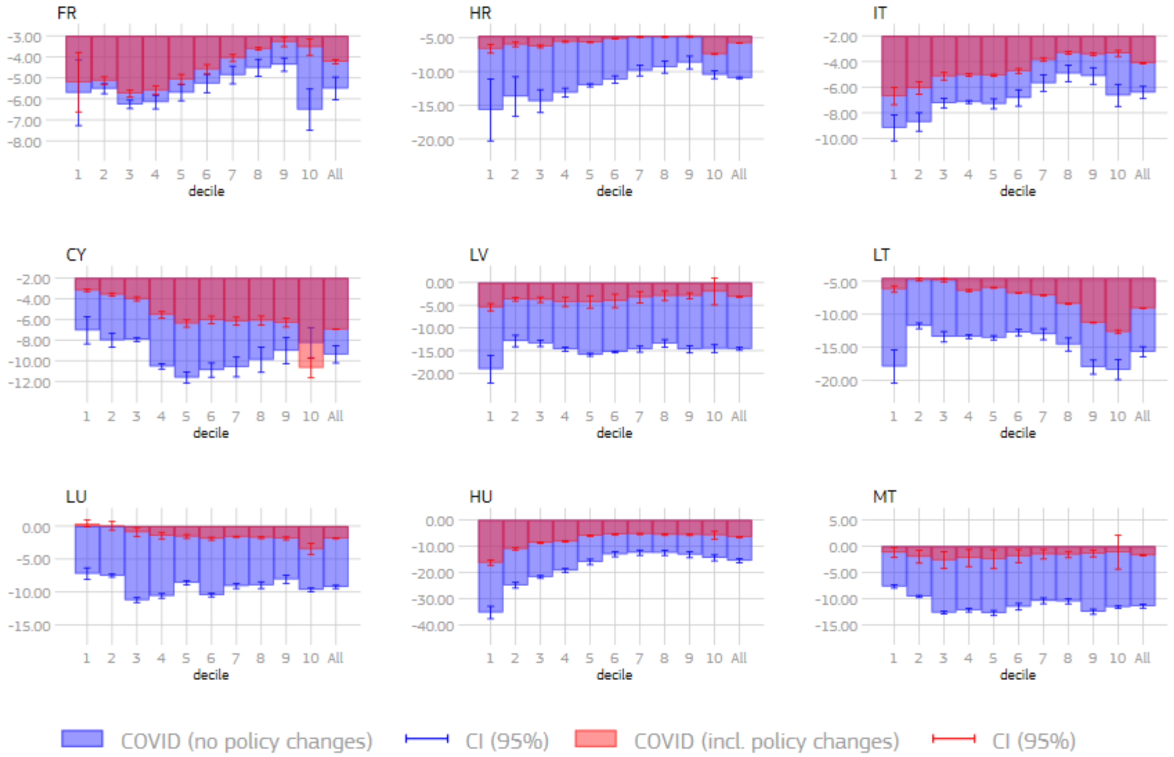

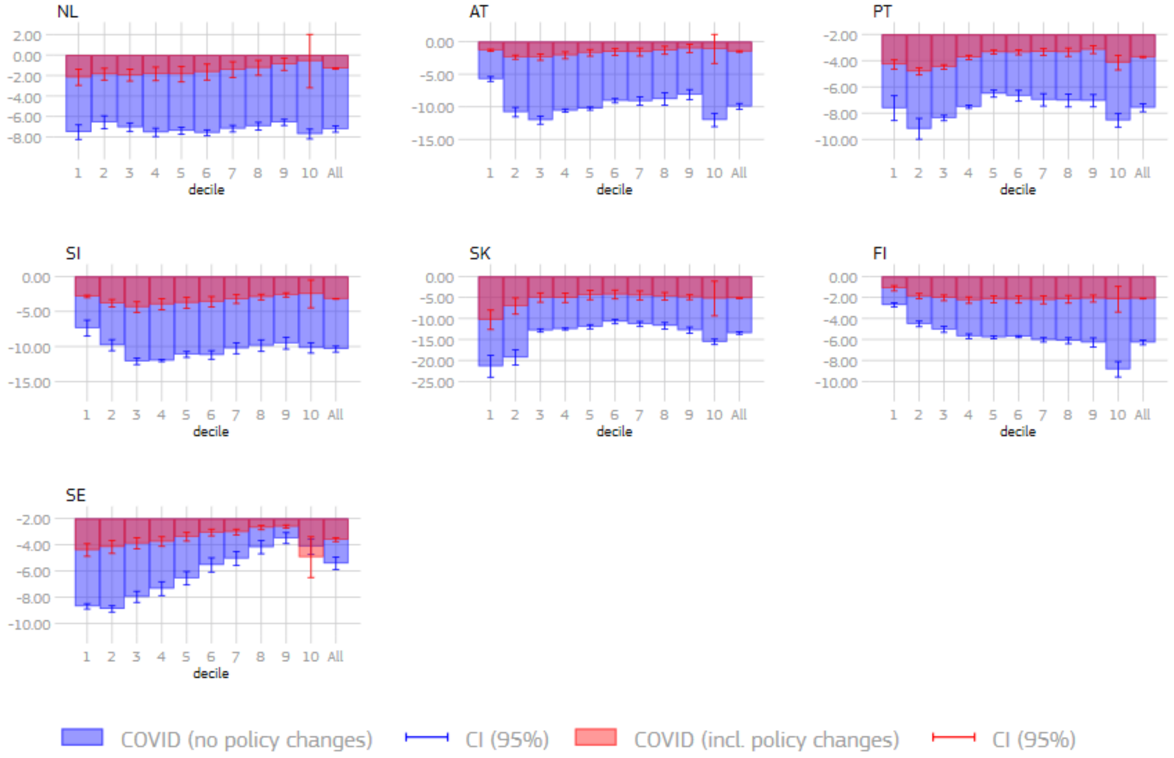

Source: own calculations using EUROMOD I2.0+.
